# Supplementary material for: Ecological and taxonomic dissimilarity in species and higher taxa of reptiles in western Mexico
Source: PeerJ. 2024 Oct 22;12:e18343. doi: 10.7717/peerj.18343 (PMC11505965; doi:10.7717/peerj.18343)
Supplement: Supplemental Information 1 [file peerj-12-18343-s001.docx]

**Supplementary Information**

Ecological and taxonomic dissimilarity in species and higher taxa of reptiles in western Mexico

Jaime Manuel Calderón-Patrón^1^, Jorge Téllez López^2^, Eréndira Patricia Canales Gómez^2^ and Karen Elizabeth Peña Joya^2^

^1^ Laboratorio de Biodiversidad de la Escuela de Ciencias, Universidad Autónoma Benito Juárez de Oaxaca, Oaxaca, México.

^2^ Laboratorio de Ecología, Paisaje y Sociedad, Centro Universitario de la Costa de la Universidad de Guadalajara, Puerto Vallarta, Jalisco, México.

Corresponding Author:

Karen Elizabeth Peña Joya ^1^

Av. Universidad 203, Delegación Ixtapa, Puerto Vallarta, Jalisco, 48280, México

Email address: karen.joya@academicos.udg.mx

Table S1. Taxonomic distinctness of the physiographic regions for Reptiles, Lizards and Snakes.

| **Physiographic region** | **Number of species** | **Taxonomic distinctness**  **Delta+** |
| --- | --- | --- |
| REPTILES | | |
| PC | 75 | 64.81 |
| SO | 63 | 56.18 |
| SJ | 68 | 52.97 |
| TV | 85 | 54.20 |
| SC | 24 | 52.96 |
| CP | 68 | 53.93 |
| TD | 23 | 49.15 |
| LIZARDS | | |
| PC | 22 | 46.83 |
| SO | 24 | 35.90 |
| SJ | 21 | 40.63 |
| TV | 29 | 37.10 |
| SC | 8 | 24.23 |
| CP | 26 | 36.42 |
| TD | 7 | 32.65 |
| SNAKES | | |
| PC | 42 | 30.62 |
| SO | 37 | 31.56 |
| SJ | 45 | 28.28 |
| TV | 54 | 30.97 |
| SC | 15 | 25.14 |
| CP | 41 | 29.72 |
| TD | 16 | 27.92 |
