# Supplementary material for: Ecological and taxonomic dissimilarity in species and higher taxa of reptiles in western Mexico
Source: PeerJ. 2024 Oct 22;12:e18343. doi: 10.7717/peerj.18343 (PMC11505965; doi:10.7717/peerj.18343)
Supplement: Supplemental Information 2 [file peerj-12-18343-s002.docx]

**Supplementary Information**

Ecological and taxonomic dissimilarity in species and higher taxa of reptiles in western Mexico

Jaime Manuel Calderón-Patrón^1^, Jorge Téllez López^2^, Eréndira Patricia Canales Gómez^2^ and Karen Elizabeth Peña Joya^2^

^1^ Laboratorio de Biodiversidad de la Escuela de Ciencias, Universidad Autónoma Benito Juárez de Oaxaca, Oaxaca, México.

^2^ Laboratorio de Ecología, Paisaje y Sociedad, Centro Universitario de la Costa de la Universidad de Guadalajara, Puerto Vallarta, Jalisco, México.

Corresponding Author:

Karen Elizabeth Peña Joya ^1^

Av. Universidad 203, Delegación Ixtapa, Puerto Vallarta, Jalisco, 48280, México

Email address: karen.joya@academicos.udg.mx

Table S2. Partitions of species beta diversity of Reptiles between pairs of physiographic regions.

| **Beta.sor** |  |  |  |  |  |  |
| --- | --- | --- | --- | --- | --- | --- |
|  | PC | SO | SJ | TV | SC | CP |
| SO | 0.6812 |  |  |  |  |  |
| SJ | 0.4126 | 0.5573 |  |  |  |  |
| TV | 0.6625 | 0.4595 | 0.3987 |  |  |  |
| SC | 0.7374 | 0.6782 | 0.5870 | 0.6514 |  |  |
| CP | 0.7622 | 0.4504 | 0.5882 | 0.3464 | 0.6739 |  |
| TD | 0.7551 | 0.6512 | 0.6484 | 0.7407 | 0.4468 | 0.7582 |
| **Beta.sim** |  |  |  |  |  |  |
|  | PC | SO | SJ | TV | SC | CP |
| SO | 0.6508 |  |  |  |  |  |
| SJ | 0.3824 | 0.5397 |  |  |  |  |
| TV | 0.6400 | 0.3651 | 0.3235 |  |  |  |
| SC | 0.4583 | 0.4167 | 0.2083 | 0.2083 |  |  |
| CP | 0.7500 | 0.4286 | 0.5882 | 0.2647 | 0.3750 |  |
| TD | 0.4783 | 0.3478 | 0.3043 | 0.3913 | 0.4348 | 0.5217 |
| **Beta.sne** |  |  |  |  |  |  |
|  | PC | SO | SJ | TV | SC | CP |
| SO | 0.0304 |  |  |  |  |  |
| SJ | 0.0302 | 0.0176 |  |  |  |  |
| TV | 0.0225 | 0.0944 | 0.0752 |  |  |  |
| SC | 0.2790 | 0.2615 | 0.3786 | 0.4430 |  |  |
| CP | 0.0122 | 0.0218 | 0.0000 | 0.0817 | 0.2989 |  |
| TD | 0.2768 | 0.3033 | 0.3440 | 0.3494 | 0.0120 | 0.2365 |
