# Supplementary material for: Ecological and taxonomic dissimilarity in species and higher taxa of reptiles in western Mexico
Source: PeerJ. 2024 Oct 22;12:e18343. doi: 10.7717/peerj.18343 (PMC11505965; doi:10.7717/peerj.18343)
Supplement: Supplemental Information 3 [file peerj-12-18343-s003.docx]

**Supplementary Information**

Ecological and taxonomic dissimilarity in species and higher taxa of reptiles in western Mexico

Jaime Manuel Calderón-Patrón^1^, Jorge Téllez López^2^, Eréndira Patricia Canales Gómez^2^ and Karen Elizabeth Peña Joya^2^

^1^ Laboratorio de Biodiversidad de la Escuela de Ciencias, Universidad Autónoma Benito Juárez de Oaxaca, Oaxaca, México.

^2^ Laboratorio de Ecología, Paisaje y Sociedad, Centro Universitario de la Costa de la Universidad de Guadalajara, Puerto Vallarta, Jalisco, México.

Corresponding Author:

Karen Elizabeth Peña Joya ^1^

Av. Universidad 203, Delegación Ixtapa, Puerto Vallarta, Jalisco, 48280, México

Email address: karen.joya@academicos.udg.mx

Table S3. Partitions of species beta diversity of Lizards between pairs of physiographic regions.

| **Beta.sor** |  |  |  |  |  |  |
| --- | --- | --- | --- | --- | --- | --- |
|  | PC | SO | SJ | TV | SC | CP |
| SO | 0.6522 |  |  |  |  |  |
| SJ | 0.4884 | 0.5556 |  |  |  |  |
| TV | 0.7647 | 0.4340 | 0.4400 |  |  |  |
| SC | 0.7333 | 0.7500 | 0.5172 | 0.6216 |  |  |
| CP | 0.7917 | 0.5200 | 0.6596 | 0.3818 | 0.7059 |  |
| TD | 0.7931 | 0.8065 | 0.7143 | 0.7778 | 0.4667 | 0.7576 |
| **Beta.sim** |  |  |  |  |  |  |
|  | PC | SO | SJ | TV | SC | CP |
| SO | 0.6364 |  |  |  |  |  |
| SJ | 0.4762 | 0.5238 |  |  |  |  |
| TV | 0.7273 | 0.3750 | 0.3333 |  |  |  |
| SC | 0.5000 | 0.5000 | 0.1250 | 0.1250 |  |  |
| CP | 0.7727 | 0.5000 | 0.6190 | 0.3462 | 0.3750 |  |
| TD | 0.5714 | 0.5714 | 0.4286 | 0.4286 | 0.4286 | 0.4286 |
| **Beta.sne** |  |  |  |  |  |  |
|  | PC | SO | SJ | TV | SC | CP |
| SO | 0.0158 |  |  |  |  |  |
| SJ | 0.0122 | 0.0317 |  |  |  |  |
| TV | 0.0374 | 0.0590 | 0.1067 |  |  |  |
| SC | 0.2333 | 0.2500 | 0.3922 | 0.4966 |  |  |
| CP | 0.0189 | 0.0200 | 0.0405 | 0.0357 | 0.3309 |  |
| TD | 0.2217 | 0.2350 | 0.2857 | 0.3492 | 0.0381 | 0.3290 |
