# Supplementary material for: Ecological and taxonomic dissimilarity in species and higher taxa of reptiles in western Mexico
Source: PeerJ. 2024 Oct 22;12:e18343. doi: 10.7717/peerj.18343 (PMC11505965; doi:10.7717/peerj.18343)
Supplement: Supplemental Information 4 [file peerj-12-18343-s004.docx]

**Supplementary Information**

Ecological and taxonomic dissimilarity in species and higher taxa of reptiles in western Mexico

Jaime Manuel Calderón-Patrón^1^, Jorge Téllez López^2^, Eréndira Patricia Canales Gómez^2^ and Karen Elizabeth Peña Joya^2^

^1^ Laboratorio de Biodiversidad de la Escuela de Ciencias, Universidad Autónoma Benito Juárez de Oaxaca, Oaxaca, México.

^2^ Laboratorio de Ecología, Paisaje y Sociedad, Centro Universitario de la Costa de la Universidad de Guadalajara, Puerto Vallarta, Jalisco, México.

Corresponding Author:

Karen Elizabeth Peña Joya ^1^

Av. Universidad 203, Delegación Ixtapa, Puerto Vallarta, Jalisco, 48280, México

Email address: karen.joya@academicos.udg.mx

Table S4. Partitions of species beta diversity of Snakes between pairs of physiographic regions.

| **Beta.sor** |  |  |  |  |  |  |
| --- | --- | --- | --- | --- | --- | --- |
|  | PC | SO | SJ | TV | SC | CP |
| SO | 0.6709 |  |  |  |  |  |
| SJ | 0.3333 | 0.5610 |  |  |  |  |
| TV | 0.5833 | 0.4725 | 0.3737 |  |  |  |
| SC | 0.7193 | 0.6538 | 0.6333 | 0.6812 |  |  |
| CP | 0.7349 | 0.4103 | 0.5581 | 0.3263 | 0.6786 |  |
| TD | 0.6897 | 0.5472 | 0.6066 | 0.7143 | 0.4194 | 0.7544 |
| **Beta.sim** |  |  |  |  |  |  |
|  | PC | SO | SJ | TV | SC | CP |
| SO | 0.6486 |  |  |  |  |  |
| SJ | 0.3095 | 0.5135 |  |  |  |  |
| TV | 0.5238 | 0.3514 | 0.3111 |  |  |  |
| SC | 0.4667 | 0.4000 | 0.2667 | 0.2667 |  |  |
| CP | 0.7317 | 0.3784 | 0.5366 | 0.2195 | 0.4000 |  |
| TD | 0.4375 | 0.2500 | 0.2500 | 0.3750 | 0.4000 | 0.5625 |
| **Beta.sne** |  |  |  |  |  |  |
|  | PC | SO | SJ | TV | SC | CP |
| SO | 0.0222 |  |  |  |  |  |
| SJ | 0.0238 | 0.0475 |  |  |  |  |
| TV | 0.0595 | 0.1212 | 0.0626 |  |  |  |
| SC | 0.2526 | 0.2538 | 0.3667 | 0.4145 |  |  |
| CP | 0.0032 | 0.0319 | 0.0216 | 0.1068 | 0.2786 |  |
| TD | 0.2522 | 0.2972 | 0.3566 | 0.3393 | 0.0194 | 0.1919 |
