# Supplementary material for: Ecological and taxonomic dissimilarity in species and higher taxa of reptiles in western Mexico
Source: PeerJ. 2024 Oct 22;12:e18343. doi: 10.7717/peerj.18343 (PMC11505965; doi:10.7717/peerj.18343)
Supplement: Supplemental Information 5 [file peerj-12-18343-s005.docx]

**Supplementary Information**

Ecological and taxonomic dissimilarity in species and higher taxa of reptiles in western Mexico

Jaime Manuel Calderón-Patrón^1^, Jorge Téllez López^2^, Eréndira Patricia Canales Gómez^2^ and Karen Elizabeth Peña Joya^2^

^1^ Laboratorio de Biodiversidad de la Escuela de Ciencias, Universidad Autónoma Benito Juárez de Oaxaca, Oaxaca, México.

^2^ Laboratorio de Ecología, Paisaje y Sociedad, Centro Universitario de la Costa de la Universidad de Guadalajara, Puerto Vallarta, Jalisco, México.

Corresponding Author:

Karen Elizabeth Peña Joya ^1^

Av. Universidad 203, Delegación Ixtapa, Puerto Vallarta, Jalisco, 48280, México

Email address: karen.joya@academicos.udg.mx

Table S5. Partitions of beta diversity of higher taxa of Reptiles between pairs of physiographic regions.

| **Beta.sorT** |  |  |  |  |  |  |
| --- | --- | --- | --- | --- | --- | --- |
|  | PC | SO | SJ | TV | SC | CP |
| SO | 0.4785 |  |  |  |  |  |
| SJ | 0.2781 | 0.3550 |  |  |  |  |
| TV | 0.4359 | 0.3065 | 0.2586 |  |  |  |
| SC | 0.6190 | 0.5049 | 0.4892 | 0.5142 |  |  |
| CP | 0.5198 | 0.3240 | 0.3654 | 0.2439 | 0.4692 |  |
| TD | 0.6236 | 0.5098 | 0.5109 | 0.5755 | 0.3594 | 0.5407 |
| **Beta.simT** |  |  |  |  |  |  |
|  | PC | SO | SJ | TV | SC | CP |
| SO | 0.35461 |  |  |  |  |  |
| SJ | 0.18675 | 0.29787 |  |  |  |  |
| TV | 0.39560 | 0.20567 | 0.22289 |  |  |  |
| SC | 0.20000 | 0.21538 | 0.09231 | 0.07692 |  |  |
| CP | 0.41781 | 0.31206 | 0.32192 | 0.15068 | 0.13846 |  |
| TD | 0.19048 | 0.20635 | 0.11111 | 0.17460 | 0.34921 | 0.23810 |
| **Beta.sneT** |  |  |  |  |  |  |
|  | PC | SO | SJ | TV | SC | CP |
| SO | 0.12390 |  |  |  |  |  |
| SJ | 0.09133 | 0.05718 |  |  |  |  |
| TV | 0.04029 | 0.10083 | 0.03573 |  |  |  |
| SC | 0.41905 | 0.28947 | 0.39687 | 0.43725 |  |  |
| CP | 0.10197 | 0.01199 | 0.04347 | 0.09322 | 0.33073 |  |
| TD | 0.43314 | 0.30345 | 0.39981 | 0.40091 | 0.01017 | 0.30257 |
