# Supplementary material for: Ecological and taxonomic dissimilarity in species and higher taxa of reptiles in western Mexico
Source: PeerJ. 2024 Oct 22;12:e18343. doi: 10.7717/peerj.18343 (PMC11505965; doi:10.7717/peerj.18343)
Supplement: Supplemental Information 6 [file peerj-12-18343-s006.docx]

**Supplementary Information**

Ecological and taxonomic dissimilarity in species and higher taxa of reptiles in western Mexico

Jaime Manuel Calderón-Patrón^1^, Jorge Téllez López^2^, Eréndira Patricia Canales Gómez^2^ and Karen Elizabeth Peña Joya^2^

^1^ Laboratorio de Biodiversidad de la Escuela de Ciencias, Universidad Autónoma Benito Juárez de Oaxaca, Oaxaca, México.

^2^ Laboratorio de Ecología, Paisaje y Sociedad, Centro Universitario de la Costa de la Universidad de Guadalajara, Puerto Vallarta, Jalisco, México.

Corresponding Author:

Karen Elizabeth Peña Joya ^1^

Av. Universidad 203, Delegación Ixtapa, Puerto Vallarta, Jalisco, 48280, México

Email address: karen.joya@academicos.udg.mx

Table S6. Partitions of beta diversity of higher taxa of Lizards between pairs of physiographic regions.

| **Beta.sorT** |  |  |  |  |  |  |
| --- | --- | --- | --- | --- | --- | --- |
|  | PC | SO | SJ | TV | SC | CP |
| SO | 0.3953 |  |  |  |  |  |
| SJ | 0.2656 | 0.3109 |  |  |  |  |
| TV | 0.4370 | 0.2222 | 0.2800 |  |  |  |
| SC | 0.6044 | 0.5610 | 0.4815 | 0.5227 |  |  |
| CP | 0.4646 | 0.3220 | 0.3675 | 0.2419 | 0.5250 |  |
| TD | 0.5870 | 0.5422 | 0.5122 | 0.5506 | 0.4222 | 0.5062 |
| **Beta.simT** |  |  |  |  |  |  |
|  | PC | SO | SJ | TV | SC | CP |
| SO | 0.3500 |  |  |  |  |  |
| SJ | 0.2034 | 0.3051 |  |  |  |  |
| TV | 0.4242 | 0.1833 | 0.2373 |  |  |  |
| SC | 0.1818 | 0.1818 | 0.0455 | 0.0455 |  |  |
| CP | 0.4138 | 0.3103 | 0.3621 | 0.1897 | 0.1364 |  |
| TD | 0.1739 | 0.1739 | 0.1304 | 0.1304 | 0.4091 | 0.1304 |
| **Beta.sneT** |  |  |  |  |  |  |
|  | PC | SO | SJ | TV | SC | CP |
| SO | 0.0453 |  |  |  |  |  |
| SJ | 0.0622 | 0.0058 |  |  |  |  |
| TV | 0.0128 | 0.0389 | 0.0427 |  |  |  |
| SC | 0.4226 | 0.3792 | 0.4360 | 0.4773 |  |  |
| CP | 0.0508 | 0.0117 | 0.0055 | 0.0523 | 0.3886 |  |
| TD | 0.4130 | 0.3683 | 0.3818 | 0.4201 | 0.0131 | 0.3757 |
