# Supplementary material for: Ecological and taxonomic dissimilarity in species and higher taxa of reptiles in western Mexico
Source: PeerJ. 2024 Oct 22;12:e18343. doi: 10.7717/peerj.18343 (PMC11505965; doi:10.7717/peerj.18343)
Supplement: Supplemental Information 7 [file peerj-12-18343-s007.docx]

**Supplementary Information**

Ecological and taxonomic dissimilarity in species and higher taxa of reptiles in western Mexico

Jaime Manuel Calderón-Patrón^1^, Jorge Téllez López^2^, Eréndira Patricia Canales Gómez^2^ and Karen Elizabeth Peña Joya^2^

^1^ Laboratorio de Biodiversidad de la Escuela de Ciencias, Universidad Autónoma Benito Juárez de Oaxaca, Oaxaca, México.

^2^ Laboratorio de Ecología, Paisaje y Sociedad, Centro Universitario de la Costa de la Universidad de Guadalajara, Puerto Vallarta, Jalisco, México.

Corresponding Author:

Karen Elizabeth Peña Joya ^1^

Av. Universidad 203, Delegación Ixtapa, Puerto Vallarta, Jalisco, 48280, México

Email address: karen.joya@academicos.udg.mx

Table S7. Partitions of beta diversity of higher taxa of Snakes between pairs of physiographic regions.

| **Beta.sorT** |  |  |  |  |  |  |
| --- | --- | --- | --- | --- | --- | --- |
|  | PC | SO | SJ | TV | SC | CP |
| SO | 0.4719 |  |  |  |  |  |
| SJ | 0.2200 | 0.3837 |  |  |  |  |
| TV | 0.3780 | 0.3591 | 0.2315 |  |  |  |
| SC | 0.5887 | 0.5044 | 0.5111 | 0.5278 |  |  |
| CP | 0.5054 | 0.3418 | 0.3667 | 0.2381 | 0.4711 |  |
| TD | 0.5310 | 0.4359 | 0.4532 | 0.5405 | 0.2500 | 0.5200 |
| **Beta.simT** |  |  |  |  |  |  |
|  | PC | SO | SJ | TV | SC | CP |
| SO | 0.3733 |  |  |  |  |  |
| SJ | 0.1959 | 0.2933 |  |  |  |  |
| TV | 0.3689 | 0.2267 | 0.1959 |  |  |  |
| SC | 0.2368 | 0.2632 | 0.1316 | 0.1053 |  |  |
| CP | 0.4458 | 0.3067 | 0.3133 | 0.1325 | 0.1579 |  |
| TD | 0.1905 | 0.2143 | 0.0952 | 0.1905 | 0.2105 | 0.2857 |
| **Beta.sneT** |  |  |  |  |  |  |
|  | PC | SO | SJ | TV | SC | CP |
| SO | 0.0986 |  |  |  |  |  |
| SJ | 0.0241 | 0.0904 |  |  |  |  |
| TV | 0.0091 | 0.1324 | 0.0357 |  |  |  |
| SC | 0.3518 | 0.2413 | 0.3795 | 0.4225 |  |  |
| CP | 0.0596 | 0.0351 | 0.0534 | 0.1056 | 0.3132 |  |
| TD | 0.3406 | 0.2216 | 0.3580 | 0.3501 | 0.0395 | 0.2343 |
