# Supplementary material for: Ecological and taxonomic dissimilarity in species and higher taxa of reptiles in western Mexico
Source: PeerJ. 2024 Oct 22;12:e18343. doi: 10.7717/peerj.18343 (PMC11505965; doi:10.7717/peerj.18343)
Supplement: Supplemental Information 8 [file peerj-12-18343-s008.docx]

**Supplementary Information**

Ecological and taxonomic dissimilarity in species and higher taxa of reptiles in western Mexico

Jaime Manuel Calderón-Patrón^1^, Jorge Téllez López^2^, Eréndira Patricia Canales Gómez^2^ and Karen Elizabeth Peña Joya^2^

^1^ Laboratorio de Biodiversidad de la Escuela de Ciencias, Universidad Autónoma Benito Juárez de Oaxaca, Oaxaca, México.

^2^ Laboratorio de Ecología, Paisaje y Sociedad, Centro Universitario de la Costa de la Universidad de Guadalajara, Puerto Vallarta, Jalisco, México.

Corresponding Author:

Karen Elizabeth Peña Joya ^1^

Av. Universidad 203, Delegación Ixtapa, Puerto Vallarta, Jalisco, 48280, México

Email address: karen.joya@academicos.udg.mx

Table S8. Correlations between total beta diversity (Beta.sor), replacement (Beta.sim) and richness differences (Beta.sne) from the seven physiographic regions of the state.

|  | Beta.sor | Beta.sim | Beta.sne |
| --- | --- | --- | --- |
| Reptiles | | | |
| *Correlation coefficient* | 0.88571 | 0.82689 | 0.85844 |
| *p-value* | 0.00000 | 0.00000 | 0.00000 |
| Lizards | | | |
| *Correlation coefficient* | 0.77013 | 0.61257 | 0.74026 |
| *p-value* | 0.00004 | 0.00316 | 0.00012 |
| Snakes | | | |
| *Correlation coefficient* | 0.93117 | 0.79883 | 0.85325 |
| *p-value* | 0.00000 | 0.00001 | 0.00000 |
